# EU’s Ordering of COVID-19 Vaccine Doses: Political Decision-Making under Uncertainty

**DOI:** 10.3390/ijerph18042169

**Published:** 2021-02-23

**Authors:** Werner Gleißner, Florian Follert, Frank Daumann, Frank Leibbrand

**Affiliations:** 1 Faculty of Business and Economics, Technical University Dresden, 01069 Dresden, Germany; 2 Future Value Group AG, 70771 Leinfelden-Echterdingen, Germany; 3 Faculty of Management, Seeburg Castle University, 5201 Seekirchen am Wallersee, Austria; florian.follert@uni-seeburg.at; 4 Faculty of Social and Behavioural Sciences, Friedrich Schiller University, 07743 Jena, Germany; frank.daumann@uni-jena.de; 5 Institut für Angewandte Wirtschaftsforschung und Wirtschaftsberatung, 96135 Stegaurach, Germany; frank.leibbrand@ngi.de

**Keywords:** COVID-19, decision-making, uncertainty, vaccine, political economy, public health

## Abstract

Worldwide, politicians, scientists, and entrepreneurs are operating under high uncertainty and incomplete information regarding the adequacy of measures to deal with the COVID-19 pandemic. It seems indisputable that only widespread and global immunity can bring normalization to social life. In this respect, the development of a vaccine was a milestone in pandemic control. However, within the EU, especially in Germany, the vaccination plan is increasingly faltering, and criticism is growing louder. This paper considers the EU’s political decision in general and the decisions of the German government to procure vaccine doses against the background of modern economics as a decision under uncertainty and critically analyzes the process.

## 1. Introduction

Given the ongoing “hard” lockdown and its high costs [1], criticism of the German government’s vaccination strategy is increasing among the German public (e.g., [2]), especially since other countries have already provided a higher proportion of their population with vaccine against the SARS-CoV-2 virus, as the following Table 1 (as of 14 February 2021) illustrates.

Israel, the UK, and the United States have significantly increased vaccination relative to population by mid-February 2021. This is leading to intense questioning in the political debate about what went wrong in the process of procurement and providing vaccines to the population. In this context, the German government, especially the Federal Minister of Health, Jens Spahn, was sharply criticized in public for the low order of COVID-19 vaccines compared to other countries. The ordering process was not carried out by the federal government—among other things, probably to avoid creating the impression of so-called “vaccination nationalism”—but by the EU. The justification given is that:

- More vaccine doses were procured from several suppliers than would have been necessary for the EU population,
- The vaccines from BioNTech/Pfizer (Mainz, Germany resp. New York City, NY, USA) and Moderna (Cambridge, MA, USA), which were procured in relatively small quantities, were relatively expensive, and
- At the time of the procurement decision, it was uncertain which supplier would be able to deliver an approved vaccine and when.

Our focus is not on the phase of a faster and/or more vigorous vaccination start. An assessment of this phase would require much more information such as the approval date of the vaccine, the pre-production status on the part of the manufacturer at the time of the (emergency) approval, availability of production facilities from the pre-corona period (this would also be a policy failure), export restrictions or data exchange and, if necessary, the willingness of the population to be vaccinated at a later date, which are currently not publicly available with sufficient precision.

We argue in the longer term that with a different ordering strategy, the end to the lockdown period and herd immunity could have been achieved at an earlier stage, thereby avoiding huge costs to the economy.

**Figure 1:**
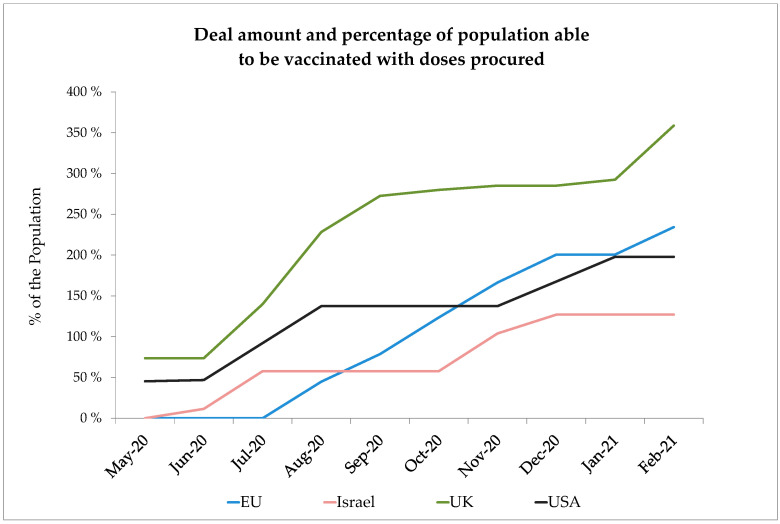
Deal amount and proportion of the population able to be vaccinated per 16 February 2021 (Sources [3,4]).

Figure 1 shows the deal amount and the proportion of the population that can be vaccinated by these doses. The calculation of the immunities per person is based on the currently expected number of doses required per person:

To point out the situation in summer 2020, which was an appropriate decision point to still react with adjustments of production capacities after procurement, Table 2 compares the orders by Israel, US, EU, and UK in or before July 2020.

This table clearly shows that the EU had not yet ordered any vaccine doses at the end of July 2020. The situation is different in the other states. For example, Israel, the United States, and the United Kingdom had initiated orders at a level with which between 35% (Israel) and 140% (United Kingdom) can be vaccinated.

Meanwhile (February 2021) the EU has placed orders of 1885 million vaccine doses for a population of almost 450 million. If we then assume a voluntary vaccination rate of 80%, this results in 2.93 immunities per person willing to be vaccinated.

The United Kingdom purchased 457 million doses of the eight most promising vaccine candidates at a very early stage. The contract with AstraZeneca, for example, was signed three months before the EU [5]. With a population of 67 million and a voluntary vaccination rate of 80%, the above assumptions result in 4.48 immunities per person, i.e., about 50% more than in the EU. Criticism is now being levelled especially at the fact that too few of the vaccines which, according to the studies, are 95% particularly effective and the fastest available, namely those from Pfizer/BioNTech and Moderna, have been procured. From the point of view of decision theory, this raises the question: Despite these attempts at explanation, is the criticism correct and do political actors bear responsibility for this wrong decision?

Already in the context of health and economic policy, the question arises to what extent politicians have disregarded economic optimality criteria. However, critics could raise the objection that ethical considerations also played a role here. Nevertheless, this argument is unlikely to apply to the procurement of a vaccine if the goal of protecting against infection, which has hitherto been propagated as seemingly absolute, remains valid for this case as well.

German virologist, Christian Drosten, emphasizes that the vaccination order cannot be evaluated from an ex-post perspective [6]. From our point of view, however, it is necessary that political decisions can also be assessed retrospectively under certain criteria, since otherwise a free space would be created that would make control by the citizen and possible consequences in future elections impossible.

Through our essay, we would like to objectify the discussion and show that, under certain conditions, a critical analysis is possible and necessary even for political decision-making under uncertainty. The central aim of this study is to examine whether the EU’s decision-making process when ordering the vaccines was appropriate and to discuss a possible better procurement strategy.

## 2. Theoretical Background: Decision-Making under Uncertainty

The COVID-19 pandemic is a global crisis that is fundamentally changing the lives of large segments of the world’s population. As it unfolds, many observers are realizing that such a complex phenomenon requires not only an epidemiological and virological dimension, but also a politico-economic perspective that critically examines political action (e.g., [7,8]). Given the classical assumptions of political and bureaucratic behavior in democracies that are worked out in detail, e.g., by Schumpeter [9], Downs [10], Tullock [11], or Niskanen [12], it appears necessary to critically examine the behavior of governments in the context of the COVID-19 pandemic. Particularly, politicians are under pressure from different lobby groups in times of crisis [7] and tend to act in a short-term oriented manner due to their high time preference rates.

Before we start our analysis, we want to point out some assumptions to keep it simple. The order policy and the order quantity decision are briefly discussed, whereby no distinction is made between the decision of the EU Commission and the decision in Germany. We also do not consider the problem of the extent to which the initially low number of vaccinations is due to (a) organizational problems, for example at the vaccination centers, or (b) initial production bottlenecks of the suppliers.

We therefore focus on the question of whether political actors, as demanding agents in the market for COVID-19 vaccines, should have ordered larger quantities from BioNTech/Pfizer and Moderna, and others even before the vaccines were approved by the EMA or the EU Commission. This concerns the assessment of a political decision under uncertainty. At the time of the decision, it was uncertain whether and when a vaccine from one of the suppliers would be approved. Equally uncertain was the efficacy of the vaccine, and other parameters of the decision problem are also uncertain, such as the economic damage that would result from an “unnecessarily delayed” vaccination of the population (e.g., through a harsher or longer lockdown).

First, it should be noted that the characteristic of all decisions, both for entrepreneurs and at the level of the state, is that the consequences of action are uncertain (e.g., [13,14,15]). It is also a normal problem that certain relevant parameters are not (yet) known due to scientific studies. It is also often overlooked in some studies that one cannot expect optimal “scientific evidence” of the state of knowledge for all decisions but must make decisions based on the real, always imperfect data situation, e.g., [16]. As long as decision-makers clearly communicate these parameters and assumptions of their decision, they can therefore not be blamed (on the Business Judgement Rule see [17,18]; on the discussion of a corresponding Political Judgment Rule [19]). It is therefore only a matter of evaluating the currently available information in the best possible way. Again, the marginal costs of information acquisition must not exceed the additional benefits (e.g., [20]), but in entrepreneurial practice this can often only be assessed by means of plausible estimates. It is obvious that the uncertainty about the data situation, and especially the quantification of risks, must also be taken into account in the decision-making process (for which adequate methods have long been developed in risk research and risk management).

In the specific example of a political decision, the costs of procuring vaccines for different order options (different quantities from different manufacturers) must essentially be weighed against the uncertain effects on the subsequent course of the pandemic. More specifically, only the cost of the right to obtain the vaccine needs to be considered, especially variable production costs do not have to be paid in the event of a delivery waiver. The economic costs due to the severity and length of the lockdown must be considered, depending on the development over time of the proportion of people who have been vaccinated. The impact of alternative procurement strategies and procurement quantities with respect to the vaccine is uncertain because the above parameters, such as the timing of a vaccine’s licensure and its efficacy or even the cost of a one-month shutdown, are uncertain. To compare the risk-benefit profile of alternative strategies for procurement, simulation models (e.g., Monte Carlo simulation, see, e.g., [21]) are used as for other decisions under uncertainty. These models capture the costs of the strategies as well as the effects on, for example, GDP (gross domestic product) or COVID-19-related deaths. The simulation models avoid spurious inaccuracies because a large representative number of risk-conditional possible future scenarios are considered. The characteristic of such methods for the well-founded preparation of decisions under uncertainty is that they show realistic development corridors of future developments depending on the decision. In this way, it is possible to state how a parameter of interest—e.g., the effect on GDP—will develop “on average” and to what extent (negative) deviations from this forecast may occur, depending on the possibilities of action. For example, one can state which damages will not be exceeded in a realistic “worst case scenario” with, e.g., 95% certainty. In this way, it is possible to select the best possible decision alternatives from those given by weighing them up, even if the information situation is imperfect. If one proceeds only from the published information, one must probably assume that a genuine quantitative risk analysis was not provided and an adequate model for the evaluation of alternative procurement strategies and procurement quantities was not used. Given the unmistakable importance of this vaccine procurement decision, this would not only be surprising, but negligent. Unfortunately, this can be interpreted as another symptom of a widespread “risk blindness” in politics (and business as well), which is probably fostered by one-dimensional advice to decision-makers. Because of the uncertain consequences, all decisions are fraught with opportunities and threats. Nevertheless, psychological research shows that people do not like to deal with such risks and do not use adequate procedures for preparing decisions under uncertainty. This opens the door to distorted perceptions by decision subjects (e.g., [22,23]).

## 3. Critical Analysis

### 3.1. A Better Decision Calculus

In our specific case of the decision on the procurement of vaccine quantities from the various suppliers, however, a special feature stands out: If we base the analysis at least on the publicly available data, we have to conclude that there is no need for a quantitative risk analysis or simulation models for the decision preparation. This is simply because the decision situation was actually trivially simple. The various sub-aspects of risk outlined above only in rudimentary form are in fact largely of subordinate practical importance. Even if a decision maker ignores the regrettably high number of fatalities caused by COVID-19 and pays attention only to the monetary effects of the “corona crisis,” each month of crisis is extremely expensive in economic terms [24]. A new study by Allianz and Euler Hermes which is available to Reuters shows that postponing vaccination schedules by five weeks could result in a loss of 90 billion euros (see, e.g., [1]). The costs of procuring a vaccine, on the other hand, are negligible. With vaccine costs per person between approximately 2 and 36 euros for the six promising suppliers, the total costs would be approximately 15 billion euros if the entire EU population had purchased from the most expensive supplier, and the investment in purchasing from all six most promising suppliers would also remain manageable [25]. Of course, in this context, it must also be taken into account at which level any cost and benefit components are to be located. However, based on the aid programs that have been set up, for example at the federal level, it can be assumed that the negative effects on the federal budget of delayed vaccination far outweigh the expenditure for vaccination.

The obvious naïve procurement strategy as a starting point would be the following: One buys so much vaccine from all promising vaccine suppliers that one could already vaccinate the entire population of the European Union or at least all persons willing to be vaccinated with the vaccine quantities of each individual supplier. The costs of such a vaccination strategy would be in the low double-digit billion range and would be negligible in view of the economic damage caused by an unnecessary prolongation of the pandemic for at least more than one month. At the time of the decision, it would probably not have been necessary to calculate the full production costs, but only the costs for the “options” for obtaining the vaccine in the event of approval. Additionally, the manageable investment for the outlined vaccination strategy, procurement of the complete quantity from all suppliers, would ultimately not be a “loss”. Of course, the EU could sell unneeded and thus surplus vaccine doses from its purchase program to other countries. Obviously, there will be a high demand for vaccines over a long period of time—and thus the risks of the outlined procurement strategy are low. True decision-making under uncertainty will lead to even better results as the above discussed naive strategy.

Of course, when choosing a strategy of this kind, the reaction of other consumers must also be kept in mind. In order to successfully implement the buying strategy, you need a time head start over the other buyers. If this advantage cannot be achieved due to the complex decision-making structures and there is therefore a simultaneous demand for the vaccine by several consumers, a price impact is more likely. Obviously, it is the case that in an outbid competition the EU is superior to most of the other buyers because of its economic strength and can outdo them, even though such behavior does not necessarily meet the highest moral standards.

### 3.2. Counterarguments and Discussion

#### 3.2.1. Hindsight Bias

Criticism concerning the EU’s vaccine procurement policy could be countered by political actors (and certainly also by scientists) with the argument that this decision is only being criticized because procurement problems have arisen that could not have been foreseen in this way. However, we want to counter this argument: Basically, the quality of a decision under uncertainty cannot be judged by the ex-post result. This is due to the fact that random influences—luck or bad luck—also determine the later result. The greater the risk, the greater the potential influence of random effects. Thus, only the information available at the time of the decision or that can be obtained at reasonable cost is relevant for the assessment of a decision.

The criticism of the EU decision outlined above is not based on the result that can now be ascertained, i.e., the shortage of vaccines. With the information available last year, based on which other countries also made their procurement decisions, a different conclusion should have been reached. The information relevant to this is outlined in Section 2. Of course, potentially useful information was not known for certain during the EU’s negotiation with vaccine suppliers. It was not known which vaccine manufacturer’s vaccine would receive approval and what efficacy they would achieve. However, this is precisely the characteristic of decision-making under uncertainty, that, e.g., every entrepreneur must deal with every day.

#### 3.2.2. Production Capacities and Production Processes

One argument often used by politicians to justify their failure to order enough quantities is that the manufacturers BioNTech/Pfizer and Moderna had a new technology, the consequences of which could hardly be assessed, combined with high procurement prices, and that the BioNTech vaccine in particular was difficult to handle [26].

However, the current problems with vaccine production capacities are less relevant for the decision regarding the amount of vaccine to be ordered last year. For our naïve solution of vaccine procurement, it is not relevant that every ordered quantity can also be delivered, so that we have the following relation:

*Q_d_* = min(*Q_o_*, *Q_p_*) (1)

where *Q_d_* = Quantity delivered, *Q_o_* = Quantity ordered, and *Q_p_* = Quantity produced.

It should be pointed out that the willingness to pay is a function of subjective preferences and therefore for the value of a good. Hence, it is in the national interest to order a sufficient quantity (and if, for example, the USA orders more consistently and more than the EU, which is more reticent, this is to the disadvantage of EU citizens). It is quite rational for each state to look after the interests of its own citizens first—this is also compatible with the legal mandate of the respective government and members of parliament. It would presumably also not be communicable to the EU population that one renounces vaccines that are in principle available—and accepts additional corona deaths—so that more can be vaccinated in other states. To put it clearly: the less vaccine is ordered for the EU, the more corona-related deaths can be expected. Even when production capacity is scarce, it is in the interest of EU citizens to secure as much of the scarce production capacity as possible.

States are not companies and are in many ways slower and less efficient than companies, although they certainly have their weaknesses in dealing with risks. For this reason, a state supply of goods can only ever be a “stopgap” solution if a private-sector solution for public goods—such as defense—is practically unsuccessful. In the case of a market solution, by the way, the EU could have ordered more vaccine doses without any problems or risks, because many people would certainly have been prepared to pay 50 or 100 euros extra for the procurement of the “full vaccine program”. This would, of course, have enabled more vaccines to be procured and the number of corona deaths to be reduced. However, benefits to those more able and willing to pay probably seem unacceptable due to the desire for “equality” (so we “sacrifice” human lives to realize the desire for more equality).

The crucial point is the following: the methods developed in modern economics for making decisions under uncertainty are equally relevant to government agencies and businesses. This is not about profit optimization. Economics explains how, given scarce resources, the goals set can best be achieved even in the face of uncertainty. What goal the government sets is fundamentally open here and normative to be decided from the government’s point of view. Insofar, we understand “economics” as a general method to analyze human behavior (see already [27]).

What we want to emphasize is the following: The state has made the wrong decision with respect to its goals, regardless of whether it is the number of deaths or the economic damage. Contrary to the typical public perception of economics, rational decision-making is not about business or profit maximization per se (e.g., [28,29,30]). It is, as mentioned, about methods to achieve subjective goals as well as possible under uncertainty, e.g., [31,32,33]). This should also be the task of the political decision-makers. In the specific case under discussion, the state would clearly have had better options for action.

#### 3.2.3. Time of Decision, Uncertainty, and Incomplete Information

It can be seen that countries that have entered into binding contractual agreements with vaccine manufacturers at a later date also tend to be supplied at a later date. With limited production capacities, it is rational for vaccine manufacturers to no longer offer binding quantity commitments (and dates) to demanders as soon as the production capacity available at a future delivery date has already been planned by earlier binding vaccine orders.

Therefore, the order date is also a critical variable that determines the availability of vaccines in a state. The reason given for the relatively late order date compared to other countries, such as the USA or Israel, is that the data situation was too uncertain at earlier times, e.g., in summer 2020. It is true that as data on progress in research and development process of individual vaccines improves, the risk of ordering is reduced. Indeed, the probability that a vaccine already in clinical phase 3 will receive approval is greater than for a vaccine that is only in clinical phase 2, clinical phase 1 or even in the preclinical phase. On the other hand, the risk increases over time because other countries place their orders earlier and the available production capacities are already occupied by their orders. This is precisely the consequence of the EU’s relatively late vaccine ordering, as was made clear, for example, in the dispute over AstraZeneca’s delivery cuts in January 2021 (the UK, which ordered earlier and was apparently able to obtain contractually clearer commitments from AstraZeneca, was less affected than the EU in the reductions in vaccine deliveries resulting from the production capacity limits).

It should also be noted that, as already explained in Section 2, decisions under uncertainty in the real world of politicians as well as entrepreneurs can never be based on a perfect state of information. It is crucial that the uncertainty of the data situation is included in the risk quantification itself. A poorer level of information is risk-increasing because it results in wider ranges with respect to the influencing factors relevant for the decision. In the example of vaccine ordering policy, this means that the bandwidth of the probability of success for the approval of a vaccine was certainly even larger in the summer of 2020 than in November 2020. Following Sinn [14] in case of complete uncertainty about the probability, the probability of a successful admission of a vaccine would be assumed in a range from 0% to 100%, equally distributed. Therefore, this uncertainty of the parameters can be taken into account in the decision calculus which is already known from economic research (e.g., [14]). Indeed, the evaluation of an uncertain benefit in preparing a decision is, at its core, precisely a mathematical uncertainty transformation that can also account for uncertainty about parameters. Political or business decisions under uncertainty should, as the above explanations show, not be postponed until all parameters of the decision problem have been clarified by sufficient scientific evidence—e.g., to estimate the approval probability of a vaccine with a very narrow bandwidth or even the approval by the authority has already been granted.

In the naive solution, the expected costs were essentially compared to the expected benefits in terms of corona damage saved. This already indicated that larger quantities of the promising vaccine candidates should certainly have been ordered earlier. However, decisions under uncertainty are usually about finding an exchange relationship between uncertainty and expected outcome. Abstractly formulated, it is about the evaluation/preference of distribution functions over time. Observable results on preferences are provided by the stock market. The results of the companies are characterized by some uncertainties and the participants in the stock markets almost always find a meaningful valuation of the companies. Even if the decision-making situation is not completely comparable, the basic idea is similar. Investors can be compensated for increased uncertainty by premiums, so that approaches such as Dorfleitner and Gleißner [34] could be used in an adapted way.

#### 3.2.4. Selection of Potential Suppliers and Costs

With respect to the negotiation situation in 2020, whether to buy (1) options or (2) vaccines, even if they might prove themselves after the trials, is definitely a major negotiating issue. Both would be generally possible. Apart from any uncertainty about the success of the development, which has to be considered, there remains a central difference: The actual variable production costs are of course not incurred if the vaccine does not work at all and therefore does not have to be produced.

What we do not want to discuss ex post is whether the “short list” of six promising producers drawn up by the EU was correct at the time of the decision and the information available at that time. It is clear that it would not have been possible to buy completely from 248 potential producers. The EU correctly applied a “pre-selection”. In the absence of alternative information, we have optimistically assumed here that this pre-selection was appropriate for six candidates. We have only shown that if there are still six promising candidates, the “full quantity” should have been purchased from these (if there had been 50 candidates or more, a different decision would probably have been made here).

With “only” six promising candidates—at that time, the Chinese and Russian vaccines were not likely to inspire much confidence—the costs—presumably for Germany in the low single-digit billion range—are in any case very low in view of the potential benefits, economic damage plus (possibly also convertible into monetary equivalent years of life via DALY) and, as mentioned, there is also only a very low risk in the case of an “excess order”: Too much procured order quantities can certainly be sold on—possibly even at a profit—(or given away with a discount to less wealthy countries). Ordering the stated 540 to 720 million doses of vaccine needed would certainly not be enough. When we keep in mind the probability of success of each trial and the uncertainty of available production capacity (see below), orders would have to be larger. For example, a formal model would require the following: How many vaccine doses do I need to procure to obtain the required number of vaccine doses with, say, 95% certainty? Possible capacity restrictions and the time dimension mentioned above must also be considered.

Thus, at the time of ordering, there were two sources of uncertainty with respect to each vaccine supplier, namely

- Success of the trial (and corresponding timing) and
- Available production capacity (also taking into account order quantities and restrictions of other countries, see below) and other scarce means of production.

If two restrictions must exist, the demander must order even more. It may be that a vaccine manufacturer has contractually agreed to supply the vaccine. However, because of capacity restrictions or production problems the supplier cannot deliver (which we see quite straight). Even then, it is obviously good to have capacity available from other manufacturers to mitigate this capacity constraint. Ultimately, if the contracts are adequate, the order buys access to the production capacity. It should be noted here that the expansion of production capacity should of course have been included in the contractual agreements. Moreover, the basic economic principle of supply and demand applies. vaccine manufacturers create more supply capacity when contractually secured demand increases—in other words, when there is a higher order volume. Larger order volumes would therefore presumably have led to higher capacity even without additional contractual obligations. This is exemplified by BioNTech/Pfizer: the CEO has expressed his amazement at the “restrained” ordering policy of the EU [35]. We now see that due to the EU’s late and belated order, capacity is only now being expanded—even with the consequence that vaccine production is temporarily reduced due to the expansion of plants, which explains the acute vaccine shortages at the present time. This acute problem is also a consequence of the wrong order quantity policy of the EU.

In particular, it can be seen that reordering is quite difficult in this environment. In September 2020, the company BioNTech, with capital from the federal government, had taken over the plant, intended for a production capacity of 750 million doses, from the pharmaceutical company Novartis (Basel, Switzerland) and subsequently rebuilt it. Despite the best support in the approval processes, production cannot start until February 2021. Building up production capacity takes the time of five to six months. Simply converting a plant that is already producing vaccines, as is done for example at the plant of the US pharmaceutical company Baxter in Halle, Westphalia, also takes at least three months. Due to these time delays inherent in production it becomes clear how important it is to order sufficient quantities in good time at the time of the initial decision on the production facilities.

#### 3.2.5. Ethical Dimension

The question of the “ethical dimension” or the pursuit of national interests is also not an argument against a larger order quantity recommended by us. We do not assume that the conscious decision to order fewer than the necessary vaccine doses—and thus to accept, for example, an additional 100,000 deaths in the EU—would be necessary or accepted by the population if it reduced 100,000 deaths in other countries. First of all, the individual responsibility of the respective government and the orientation towards the interests of its own population apply here (especially since, due to the extraordinarily high age, vaccines are probably even particularly important in Europe), which seems to be understandable already from an agency theoretical point (e.g., [19,36]). Furthermore, this assessment is also supported by a recent expert opinion by Volker Erb [37], a German legal scholar, who considers a failure to maximize the acceleration of vaccine procurement to be a criminal act by the federal government. In particular, Erb [37] does not consider possible criticism of “vaccine nationalism” by the media or other governments as a sufficient counterargument.

Other states, which are now able to vaccinate more quickly, have precisely pushed through their own interests and it is therefore extremely disadvantageous if the EU does not act in this way. This can also be clearly seen in the “America First policy” of the USA, which is recognizable here. In addition to an earlier order and the purchase of larger quantities, additional measures have been taken and it has been decided that vaccines produced in America will only be distributed to Americans. Even the Canadians must source their vaccines from Europe. The EU is obviously much less able or willing to enforce the interests of its own citizens. The fear of being accused of a “nationalistic” policy is obviously detrimental here. Apart from that, what has been explained above applies: If the EU had ordered more than is needed in vaccine, the “surplus” could, of course, be made available to other countries without any problem. This, too, should at least mitigate the ethical problem.

## 4. Conclusions

The decision to procure the vaccine quantities was a political decision under uncertainty. Political as well as business decisions under uncertainty require a quantitative risk analysis and the use of simulation models in order to assess the various alternative courses of action. In most cases, decisions under uncertainty are complex. However, the EU’s decision to procure the COVID-19 vaccine was indeed, for once, an obvious one: it would have made sense to secure from all promising potential vaccine suppliers the amount of vaccine that alone would be sufficient for the entire EU population. The cost of vaccine procurement is low considering the economic cost of each additional week of the pandemic. If this purchasing policy ultimately results in more vaccine than needed, there will certainly be buyers in other countries or booster vaccines may be needed.

As far as the publicly available information is concerned, one has to conclude that the EU Commission—and the politicians in Germany—have made a grave mistake (and it remains a mistake even if other restrictions—e.g., on vaccine production—are taken into account). We see here (once again) an example of risk blindness and lack of skills in dealing with uncertainty in important decisions. What we did not consider in our analysis is the temporal dimension. As already mentioned, it is essential that the EU has not only ordered too little, but also too late. The vaccination success of other countries is precisely because they have been much more aggressive—and arguably more mindful of the small costs and risks of vaccine ordering versus the potentially huge benefits.

The following implications can now be drawn from our study:

(1) The EU’s decision-making process is taking too long and needs to be optimized in terms of time.
(2) Important information is apparently insufficiently taken into account in the decision-making process.
(3) The decision tools used appear to be inadequate.

The EU should draw the following conclusion from this: A decision-making process must be defined which, in the event of comparable occurrences, provides for a rapid collection of all relevant available information and which is based on a comprehensive risk assessment and relevant decision-making tools. In addition, it must be ensured that such a process is carried out in a short time.

It seems important to emphasize again—especially for readers from other disciplines—that, when we use the term “economic(s)” this does not mean an orientation towards corporate profits per se. We understand it as a “*mental skill* that incorporates a special view of human behavior” [29] (p. 5) to answer the central question of humanity, namely how an individual uses his scarce resources according to his subjective preferences in a way that increases his utility (e.g., [29,30,38]). Regarding this understanding of “economics” we refer to Frank Knight [39] (p. 95) who states:

“*From a rational or scientific point of view, all practically real problems are problems in economics. The problem of life is to utilize resources ‘economically,’ to make them go as far as possible in the production of desired results. The general theory of economics is therefore simply the rationale of life—in so far as it has any rationale!*”

Moreover, this should also be expected from a democratic government, even though wrong decisions can of course also result from the fact that decision-makers are primarily guided by the expected effects on election day [10].

We must limit the results of our analysis insofar that, due to the topicality of the subject matter, we have not been able to consider every recent development in the last days. Again, the information was incomplete at the time of the first draft of the essay, and over time some conclusions could turn out to be in need of correction. Therefore, we encourage other scientists to critically accompany the process, especially by way of interdisciplinary exchange.

## Figures and Tables

**Table 1:** Vaccinations per 14 February 2021 (Source [3]).

Country	Population	Date	Total Cases Per Million	Total Deaths Per Million	Vaccinations Per Hundred
**China**	1,439,323,774	9 February 2021	70	3	2.82
**EU**	444,919,060	14 February 2021	47,222	1152	4.88
Denmark	5,792,203	14 February 2021	35,401	396	6.99
France	65,273,512	14 February 2021	53,129	1240	4.27
Germany	83,783,945	14 February 2021	27,950	777	4.95
Italy	60,461,828	14 February 2021	45,018	1548	4.96
Netherlands	17,134,873	14 February 2021	60,902	872	3.37
Spain	46,754,783	14 February 2021	65,363	1385	5.48
**Israel**	8,655,541	14 February 2021	83,690	622	74.50
**Russia**	145,934,460	10 February 2021	27,192	527	2.67
**Serbia**	6,804,596	13 February 2021	61,648	619	13.97
**UK**	67,886,004	14 February 2021	59,658	1729	23.33
**USA**	331,002,647	14 February 2021	83,505	1466	15.81

**Table 2:** Orders in or before July 2020 (Sources [4]).

Country	Population	Orders in or before July 2020	Proportion of the Population
EU	444,919,060	0	0%
Israel	8,655,541	6,000,000	35%
UK	67,886,004	190,000,000	140%
USA	331,002,647	610,000,000	92%

## Data Availability

Not applicable.
